# Structural polymorphisms in fibrillar aggregates associated with exfoliation syndrome

**DOI:** 10.1038/s41598-020-72737-6

**Published:** 2020-09-28

**Authors:** Mehdi Ghaffari Sharaf, Sara Amidian, Vineet Rathod, Andrew Crichton, Karim F. Damji, Holger Wille, Larry D. Unsworth

**Affiliations:** 1 Department of Chemical and Materials Engineering, University of Alberta, Edmonton, AB Canada; 2 Department of Biochemistry and Centre for Prions and Protein Folding Diseases, University of Alberta, Edmonton, AB Canada; 3 Department of Ophthalmology, University of Calgary, Calgary, Canada; 4 Department of Ophthalmology and Visual Sciences, University of Alberta, Edmonton, AB Canada

**Keywords:** Molecular conformation, Glaucoma

## Abstract

Exfoliation syndrome is largely considered an age-related disease that presents with fibrillar aggregates in the front part of the eye. A growing body of literature has investigated structural diversity of amyloids and fibrillar aggregates associated with neurodegenerative disease. However, in case of exfoliation syndrome, there is a dearth of information on the biophysical characteristics of these fibrils and structural polymorphisms. Herein, structural diversity of fibrils isolated from the anterior lens capsule of patients was evaluated using transmission electron microscopy techniques. It was apparent that, despite having a low sample number of different patients, there exists a wide range of fibril morphologies. As it is not precisely understood how these fibrils form, or what they are composed of, it is difficult to postulate a mechanism responsible for these differences in fibril structure. However, it is apparent that there is a wider range of fibril structure than initially appreciated. Moreover, these data may suggest the variance in fibril structure arises from patient-specific fibril composition and/or formation mechanisms.

## Introduction

Exfoliation syndrome (XFS) presents with fibrillar aggregates in many tissues throughout the body and within the eye it is largely, but not solely, located in the anterior segment^1,2^. Exfoliation materials mostly contain elastic fiber components and are thought to be formed through overproduction of elastic microfibrils and their assembly into fibrillar aggregates^3^. These aggregates can lead to cataract formation and impede aqueous humor drainage, leading to increased intraocular pressure (IOP) that causes exfoliation glaucoma. Briefly, movement of the iris sphincter region over the anterior surface of the lens capsule during physiologic pupillary motion may liberate XFS material from the capsule which then deposits in the trabecular meshwork. In addition, the exfoliation particles can act as sandpaper during iridozonular friction, leading to rupture of a weak iris pigment epithelium, pigment loss from the iris, and subsequent distribution of pigment particles within the anterior chamber^4,5^. Early electron microscopy studies yielded the presence of cross-banded fibrillar structures and filamentous subunits in a ground of amorphous aggregates of ocular tissues from XFS patients^6,7^. Studies have suggested structural similarities between amyloid fibrils and XFS material^8,9^, however, due to some inconsistence findings and limited number of investigations on the biophysical characteristics of XFS materials, the similarity between amyloid and XFS aggregates is debated^4^.

Correlating the precise similarities between the structure of XFS and amyloid fibrils is outside the scope of this study; as high-resolution studies of XFS fibril core structure would be required to properly address this question. That said, structural polymorphisms are a key characteristic of amyloid fibrils, where variations in the structure of fibrillar aggregates might be correlated with variations in disease phenotype or biological mechanisms underlying disease development^10,11^. So important is this characterization that patient-specific structural variations in amyloid fibrils have been thought to be key to developing structure-specific diagnostic agents^10,12^. Thus, amyloid polymorphisms and structural variations of fibrillar aggregates associated with neurodegenerative diseases have been extensively characterized^10,12^. However, early microscopy studies of XFS materials primarily focused on finding fibrils in different ocular structures, as opposed to precisely defining their biophysical characteristics^13^.

For other fibril forming diseases it is acknowledged that structural polymorphisms play an important role in their pathomechanism^14,15^; however, this topic seems largely unstudied for XFS. Despite the fact that conformational diversity of fibrillar aggregates might lead to different physiological consequences^16^, there are limited studies done to determine the potential diversity of XFS fibrils between patients. Previous transmission electron microscopy (TEM) analysis of XFS fibrils on human lens capsules have suggested that XFS materials are generally composed of two main types of fibrillar structures: (1) fibril diameter of ~ 20 nm and length of ~ 1 µm, with common banding patterns every ~ 25 or 50 nm (25 nm being less frequent); (2) fibril diameter of ~ 40 nm and length from 0.3 to 0.5 μm, with a less frequent banding pattern. It has been hypothesized that these fibrils form from protofilaments having a range of diameters from ~ 5 to ~ 9 nm with banding pattern of ~ 11 nm^4^. However, patient to patient variability in the structural polymorphisms of these formed fibrils has not been reported.

In this study, we confirmed the presence of XFS fibrils on human lens capsules using different microscopy techniques. Isolated XFS deposits from the anterior lens capsules of various patients, at different stages of the disease (Table 1), used TEM techniques to evaluate the structural characteristics of these fibrils. A large variety in structural features of these fibrils were observed. Despite the small sample size of patients studied a large number of differences in fibril structure was observed. It seems obvious that further structural studies need to be conducted to fully characterize these fibrillar structures.

**Table 1:** XFS samples used for TEM studies.

Sample name	Patient age	Patient sex	Amount of XFS material
L	61	Male	Low
M1	72	Male	Moderate
M2	66	Male	Moderate
M3	83	Male	Moderate
H	67	Male	Heavy

## Results and discussion

Patient specific samples of XFS materials collected from the anterior side of excised lens capsules were further characterized using stereomicroscopy, SEM and TEM techniques. It should be mentioned that the identification of XFS materials may not fully represent the population of materials present before surgery as the surgery itself may inadvertently remove some XFS materials from the capsule; this is despite the fact that standard surgical practices are employed from patient to patient and from surgeon to surgeon. In addition to this, the patient-to-patient variation in deposition patterns of XFS materials on lens capsules may result in differences in amount of XFS materials or even structural types of XFS materials present after surgery. Thus, despite being clinically diagnosed with XFS, only five out of nine tissue samples clearly had fibrils present.

In contrast to complex purification strategies used to process samples from amyloidotic organs, like the brain or liver, that may induce artifacts in their analysis^17^ the method for capturing XFS materials is relatively straightforward. Similar to previously reported methods^18^, we used a gel-loading pipette tip to remove XFS materials using a gentle mechanical force that removed aggregates without damaging underlying tissues. Thus, it is expected this approach will minimize contamination through capturing collagen fragments or addition of enzymes for degrading the lens capsule itself. Given the well-defined and significant differences in size and structure between XFS and collagen fibers^19^, it was apparent that no collagen fibers were observed in our TEM studies of XFS materials^20,21^.

### Identification of XFS materials on the lens capsule

**Figure 1:**
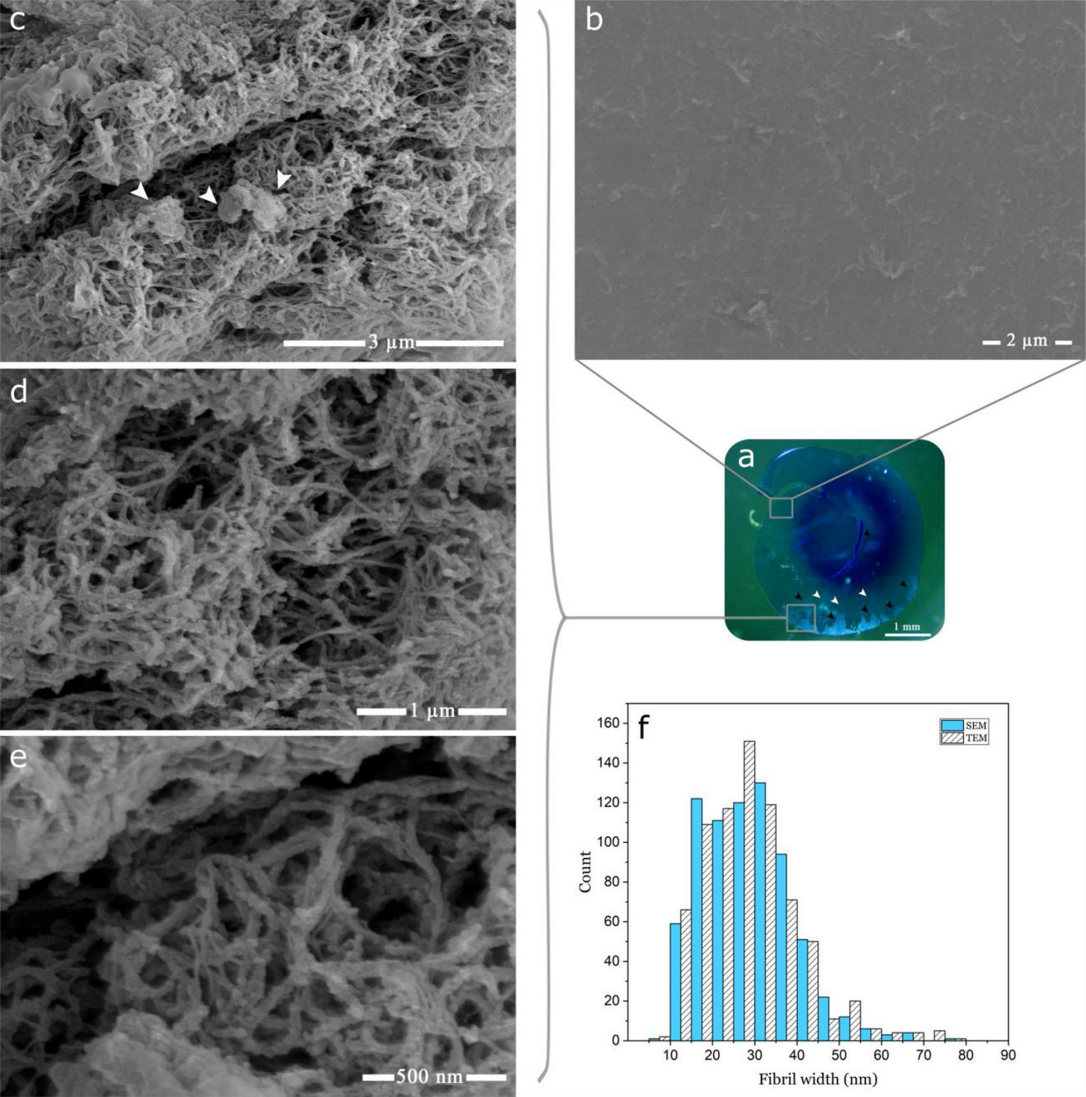
Micrographs of the anterior surfaces of lens capsules excised from XFS patients. (**a**) Lens capsule under stereomicroscope (black arrows indicate XFS deposits, white arrows indicate the junctions between intermediate and peripheral zones). (**b**) SEM micrograph of a non-XFS rich capsule location. (**c**–**e**) SEM micrographs of XFS fibrils found on the capsule surface. (**f**) Diameter distribution of XFS fibrils on the capsule surface as observed using SEM and TEM.

XFS coated human lens capsules were analyzed using light and scanning electron microscopy (SEM, Fig. 1). Stereomicroscopy was used to confirm the presence of XFS deposits on the anterior side of all lens capsules prior to subsequent processing for electron microscopy characterization. A representative stereomicroscopy result is shown in Fig. 1a for XFS deposits from a 68-year-old male patient, with a moderate level of XFS, where the lens was stained with trypan blue ophthalmic solution used during cataract surgery. Because of the deep blue color, XFS deposits were not observed clearly in the central zone of the lens capsule, however, XFS deposits were clearly observed in the peripheral zone (Fig. 1a). Junctions between intermediate (i.e. devoid of XFS materials) and peripheral zones were also observed (white arrows, Fig. 1a). The observed connecting bridge pattern between the central zone and peripheral zone is common for clinically identified XFS^2^. The intermediate clear zone, where the lens capsule is normal and mainly devoid of XFS deposits^22^, was also clearly observed in stereomicrographs, and further high-resolution SEM confirmed this area was completely devoid of XFS fibrils (Fig. 1b). This zone is believed to be created due to the rubbing of iris sphincter region over the anterior surface of the lens capsule during physiologic pupillary movement^4^.

SEM studies of XFS materials were used to confirm the presence of a loosely packed irregular meshwork of XFS fibrils on the surface of the lens capsule, as previously reported (Fig. 1c–e)^22^. The lens capsule collected from a 76-year-old female patient, with moderate XFS, was used to evaluate the XFS materials on the lens using SEM (Fig. 1b–e). Different locations of the same lens capsule were characterized (Fig. 1b–e), the results of which are similar to previously reported structures on the lens capsule. The diameter of the majority of XFS fibrils were found to be in the range of ~ 15 to ~ 40 nm (Fig. 1f). Thinner fibrils of ~ 10 nm diameter were observed that could be protofilaments, which may aggregate laterally to form thicker mature fibrils that are up to ~ 80 nm in diameter (Fig. 1e). SEM studies on XFS fibrils associated with human lens capsules have shown fibril diameter ranges of ~ 50–80 nm and ~ 35–40 nm with subunit filaments having a diameter of ~ 10 nm^22,23^. Spherical aggregates (white arrows, Fig. 1c) were observed as reported previously, where others have conjectured them to be cell fragments^23^. Because of the irregular and tangled arrangement of XFS fibrils, it is difficult to ascertain individual fibril length; we could barely identify fibrils exceeding 1 μm in length, however, different SEM studies have reported lengths ranging from ~ 3 to 5 μm^22,23^.

**Figure 2:**
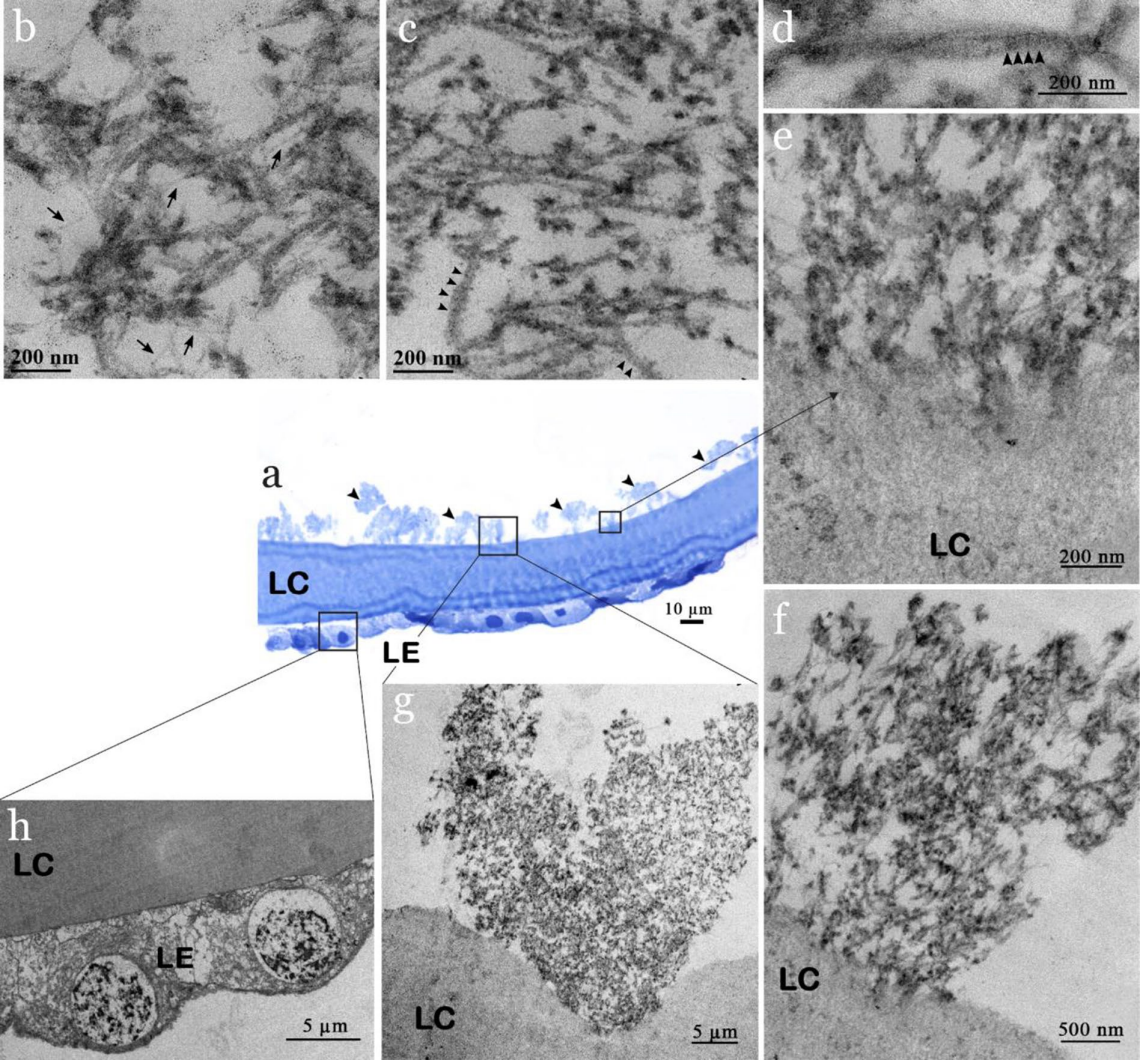
Micrographs of the cross-section of anterior lens capsules excised from XFS patients. (**a**) Light microscope image of sectioned lens capsule showing bush-like excrescence of XFS material on the surface of lens capsule (arrows). (**b**) TEM micrograph of XFS fibrils being formed through lateral binding of protofilament subunits. (**c**,**d**) XFS fibrils on the anterior lens capsule, with cross-banding pattern (arrows). (**e**) Interface contact between lens capsule and XFS fibril. (**f**,**g**) Differences in interfacial contacts with XFS materials as well as XFS macrostructure. (**h**) Lens epithelial cells on the posterior side of lens capsule (*LC* lens capsule, *LE* lens epithelium).

Using some of the same lens capsule analyzed using SEM (Fig. 1), an expected bush-like, feathery excrescence of XFS materials was also observed using light microscopy (Fig. 2a)^2^. As already seen in the SEM micrographs, an irregular orientation of non-branching XFS fibrils was also observed in TEM micrographs (Fig. 2b,c). Diameter distribution analysis on TEM micrographs showed that XFS fibrils had a similar width distribution as observed using SEM, where the majority of fibrillar aggregates had diameters ranging from ~ 15 to ~ 40 nm (Fig. 1f). Protofilaments with a diameter ~ 10 nm were observed within the mature XFS matrix (arrow, Fig. 2b). These protofilament subunits are thought to laterally aggregate to form fibrils up to 80 nm in width (Figs. 1f and 2b)^24^. Previous electron microscopy studies have shown that XFS fibrils usually exhibit a periodic cross-banding pattern^2,4,25^. In this study, a banding pattern was observed in some TEM micrographs of XFS fibrils in lens capsule sections (Fig. 2c,d). The core structure of XFS fibrils has not been clearly identified, thus a molecular basis for the observed cross-banding patterns is unknown. However, collagen fibers show a highly ordered banding pattern that is due to the specific packing of tropocollagen monomers, which create regular gap and overlap regions along the fiber^26^. That said, this structure in collagen arises from a controlled fibrillogenesis process, whereas XFS fibrils have only been associated with cells that have an increased metabolism, where previous studies have shown indications of an active fibrillogenesis process^24,27^. However, it is unclear what molecular-level interactions are responsible for cross-banding patterns seen in XFS fibrils.

The epithelial cells located on the posterior side of the lens capsule were evident in both light and TEM micrographs (Fig. 2a,h). Previously reported TEM studies have shown that part of the XFS materials found on the anterior lens capsule are in fact intracapsular XFS fibrils, which emerge from the lens epithelium and migrate through the lens capsule to its surface^2,28^. However, although our data may suggest that a similar structure within the lens capsule exists (arrow, Fig. 2e,f), we focused on analyzing the fibrils exposed on the anterior side of the capsule only. Excrescences of XFS fibrils on the anterior lens capsule were also observed as expected (Fig. 2g).

### XFS materials liberated from the capsule

**Figure 3:**
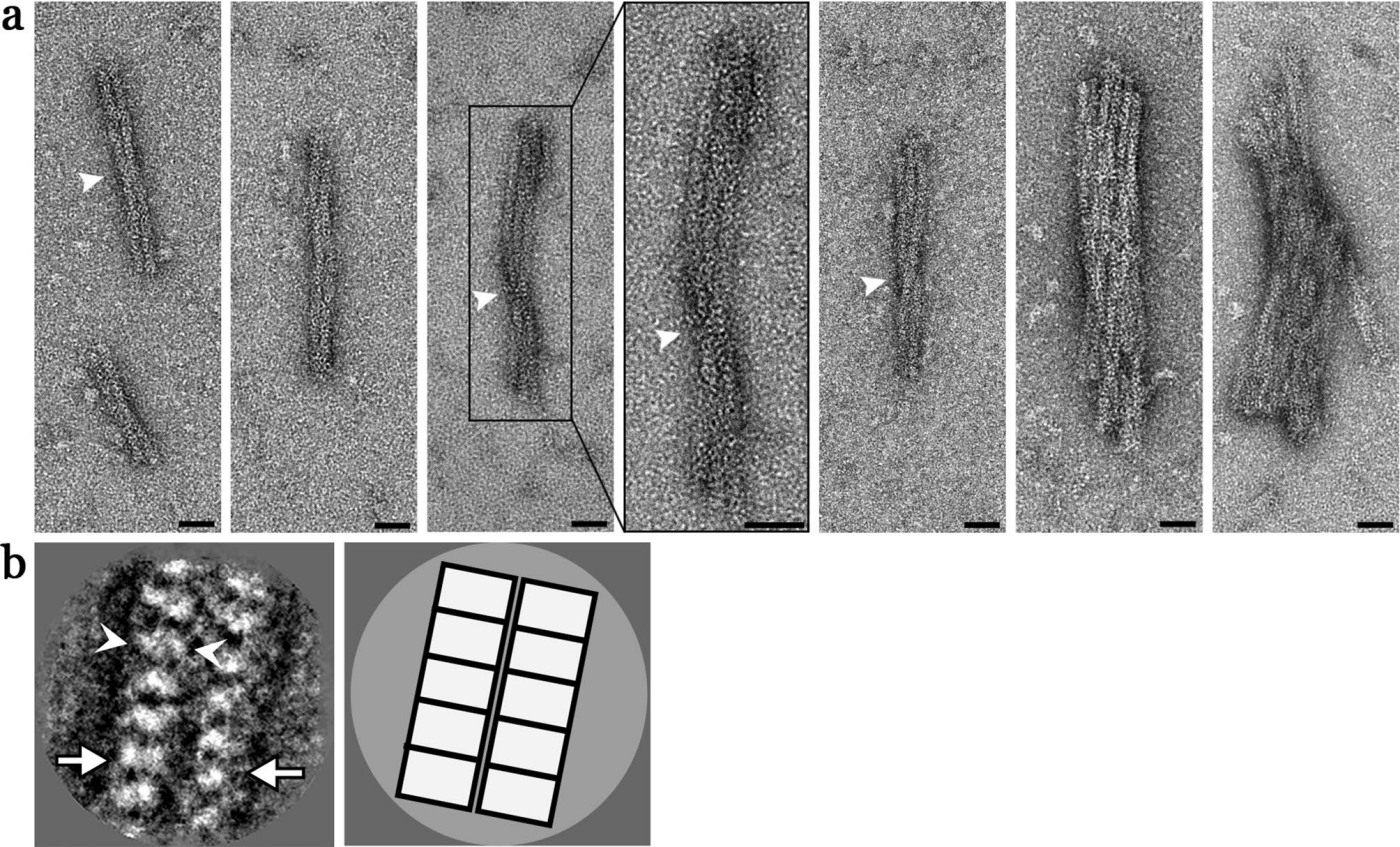
Electron micrographs of XFS fibrils from the surface of lens capsule XFS-M1. (**a**) Electron micrographs of negatively stained (using 2% UA), isolated XFS deposits from the lens capsule of a 72-year-old male patient (M1) being in moderate stages of XFS at the time of surgery. Arrows in (**a**) indicate helical twist regions (scale bars = 50 nm). (**b**) First panel: a representative 2D class average of the two protofilament fibril segments after image processing. One and two protofilament is designated by white arrowheads and arrows, respectively (scale bar = 10 nm). Second panel: a schematic illustration of the 2D class average image.

**Figure 4:**
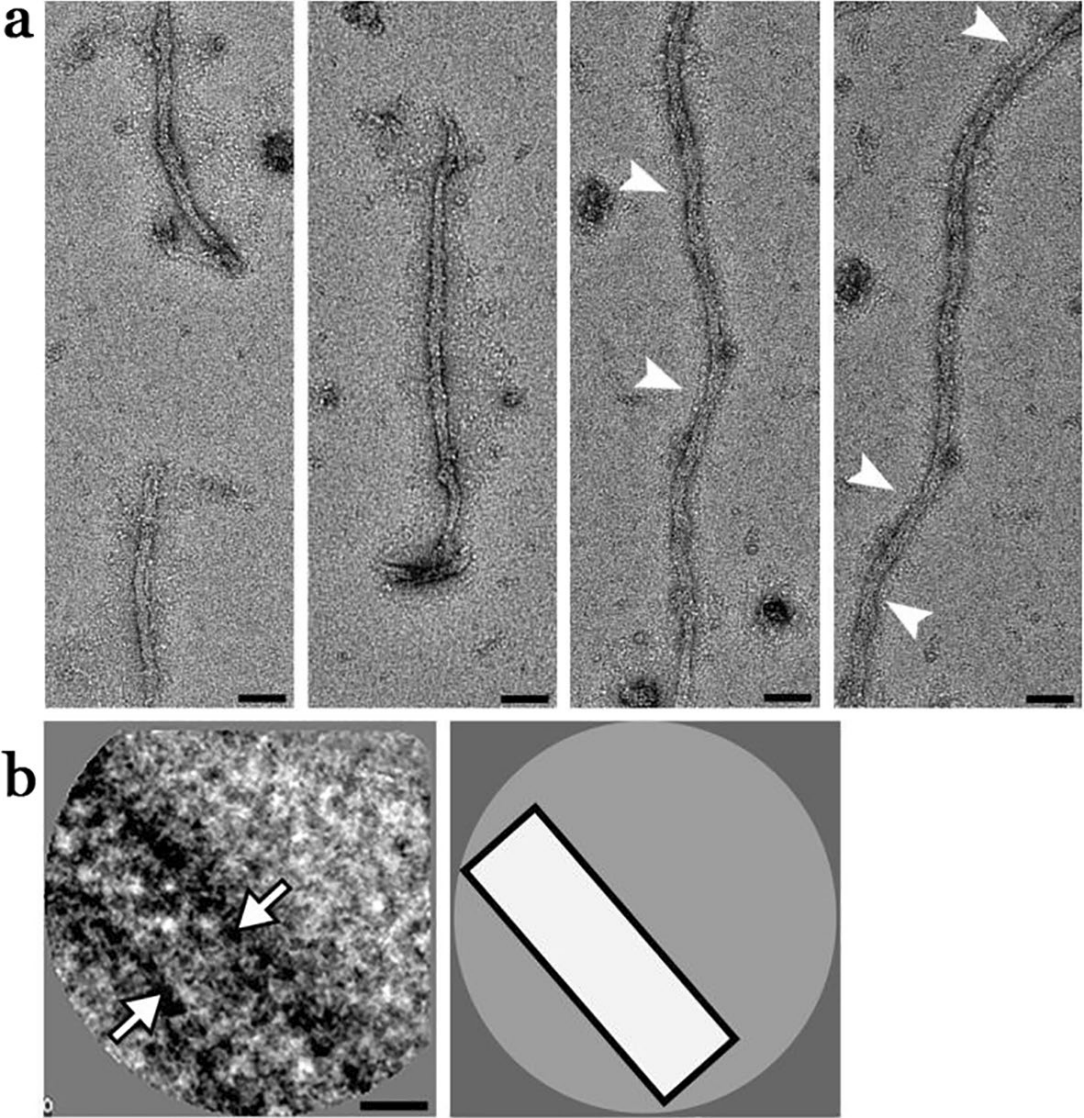
Electron micrographs of XFS fibrils from the surface of lens capsule XFS-M2. (**a**) Electronmicrographs of negatively stained (using 2% UA), isolated XFS deposits from a lens capsule of a 66-year-old male patient (M2) being in moderate stages of XFS at the time of surgery. Arrows in (**a**) indicate helical twist regions (scale bars = 50 nm). (**b**) First panel: a representative 2D class average of the fibril segments after image processing (scale bar = 10 nm). White arrows indicate the density of the fibril in the 2D class average image suggesting it is consist of one protofilament only. Second panel: a schematic illustration of the 2D class average.

**Figure 5:**
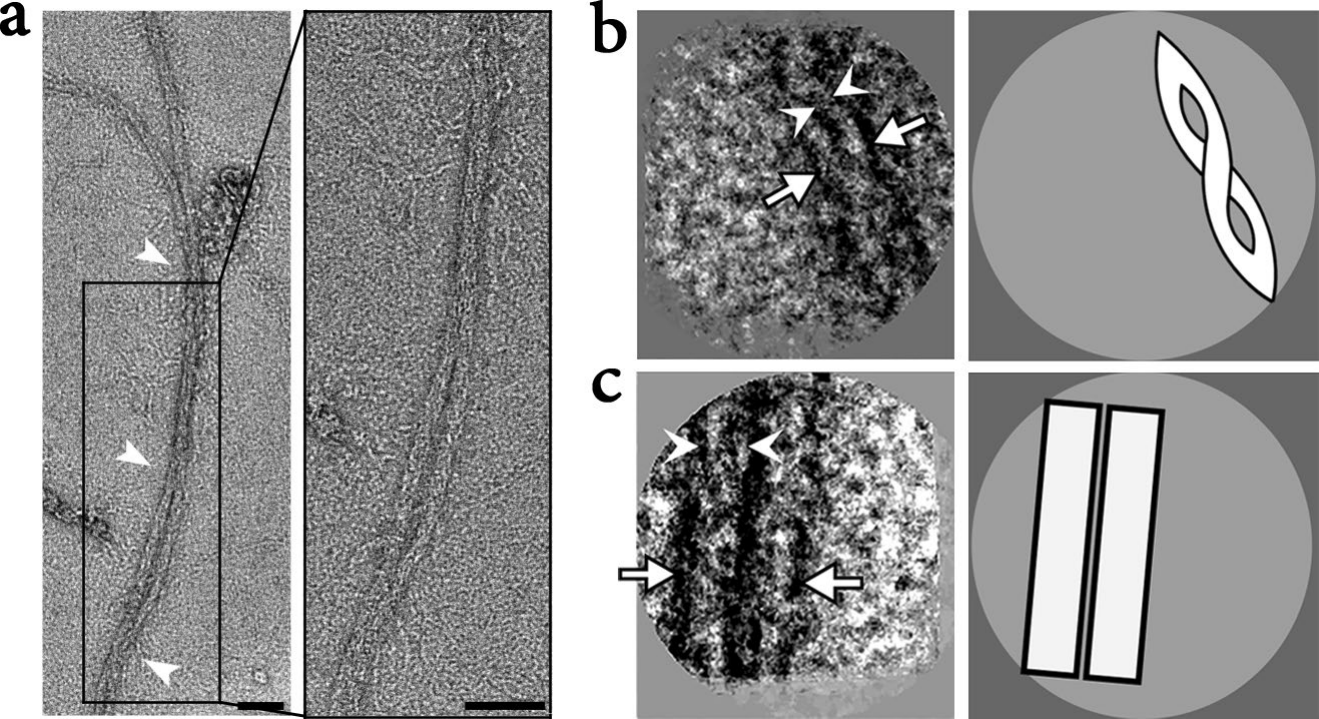
Electron micrographs of XFS fibrils from the surface of lens capsule XFS-M3. (**a**) Electron micrographs of negatively stained (using 2% UA), isolated XFS deposits from a lens capsule of an 83-year-old male patient (sample M3) being in moderate stages of XFS at the time of surgery. Arrows in (**a**) indicate helical twist regions (scale bars = 50 nm). (**b**) First panel: a representative 2D class average of the 10 nm filaments displaying two protofilaments. One and two protofilaments are designated by white arrowheads and arrows, respectively. Second panel: a schematic illustration of the 2D class average image (scale bar = 10 nm). (**c**) First panel: 2D class average of 20 nm fibrils displaying two distinct physical entities. One and two protofilaments are designated by white arrowheads and arrows, respectively. Second panel: a schematic illustration of the 2D class average image (scale bar = 10 nm).

**Figure 6:**
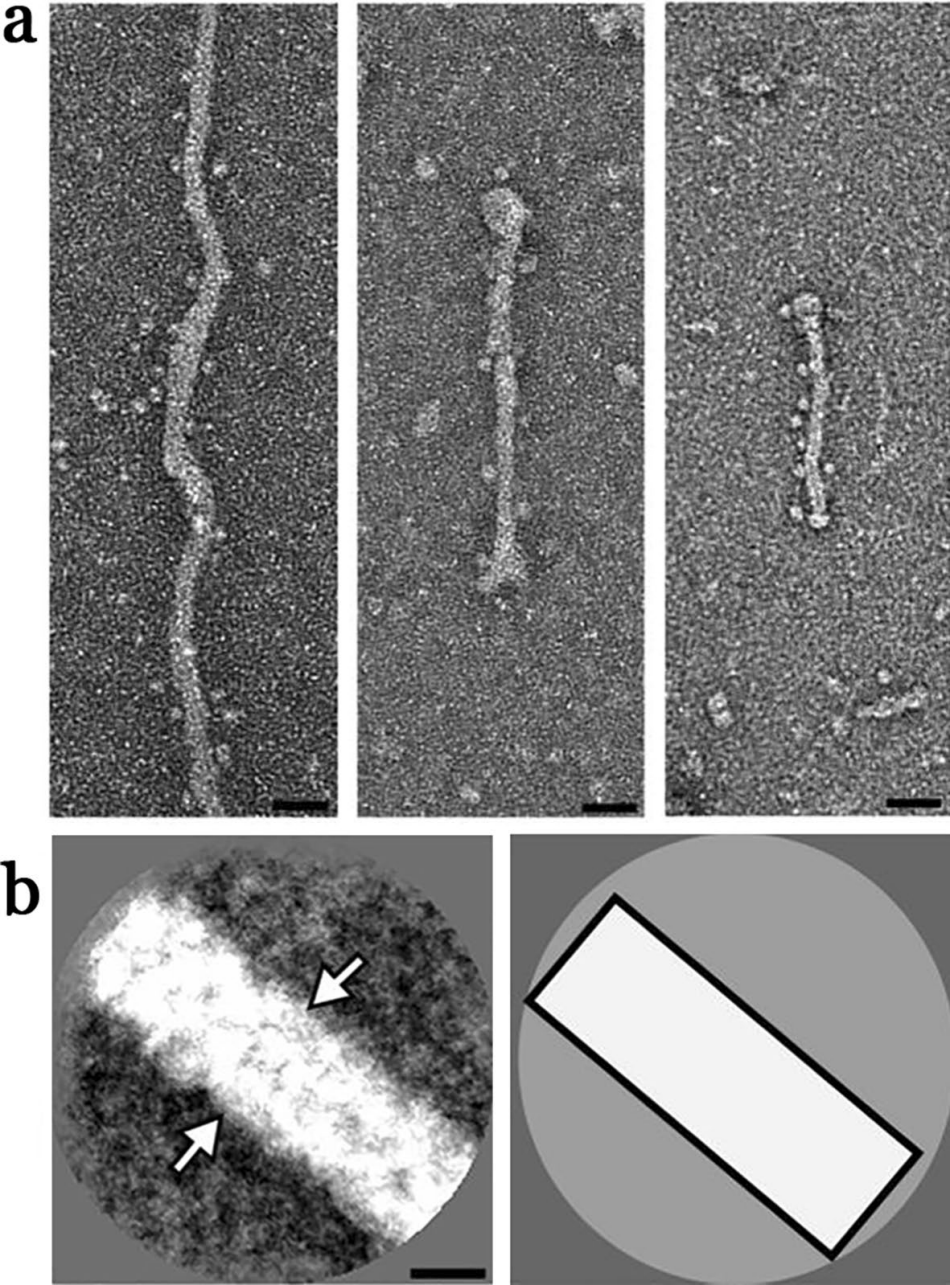
Electron micrographs of XFS fibrils from the surface of lens capsule XFS-L. (**a**) Electron micrographs of negatively stained (using 2% PTA), isolated XFS deposits from a lens capsule of a 61-year-old male patient (sample L) being in low stages of XFS at the time of surgery (scale bars = 50 nm). (**b**) First panel: a representative 2D class average of the fibril segments. White arrows indicate the density of the fibril in the 2D class average image (scale bar = 10 nm). Second panel: a schematic illustration of the 2D class average image.

**Figure 7:**
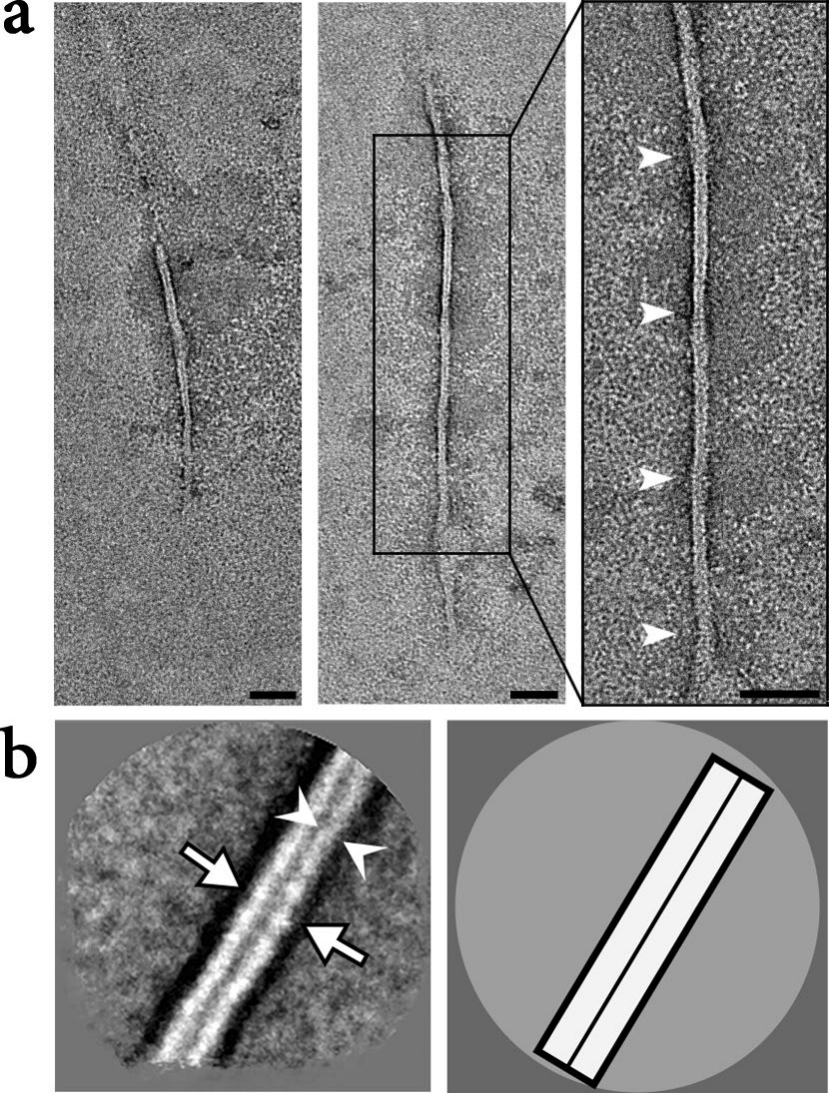
Electron micrographs of XFS fibrils from the surface of lens capsule XFS-H. (**a**) Electron micrographs of negatively stained (2% PTA), isolated XFS deposits from a lens capsule of a 67-year-old male patient (sample H) being in high stages of XFS at the time of surgery. Arrows in (**a**) indicate helical twist regions (scale bars = 50 nm). (**b**) First panel: a representative 2D class average of the fibril segments. One and two protofilaments are designated by white arrowheads and arrows, respectively (scale bar = 10 nm). Second panel: a schematic illustration of the 2D class average image.

The ultrastructure of XFS materials obtained from five clinically diagnosed patients were characterized using negative stain TEM (Figs. 3, 4, 5, 6, 7). Figures 3, 4 and 5 are stained samples from patients with moderate amounts of XFS deposits on their lens capsules. Figures 6 and 7 are from patients with low and high amount of XFS deposits, respectively. In contrast to previous results, it is apparent that the characterized XFS fibrils have a distinct morphology in each patient sample.

As seen in Fig. 3a, isolated fibrils from a 72-year-old male patient, of moderate XFS, had distinct substructures and contained two or more protofilaments. These fibrils were highly abundant on the EM grid. The width of fibrils with one, two (e.g., Fig. 3a first two panels), and three protofilaments were measured to be ~ 16, 31, and 48 nm, respectively (Table 2). As seen in the last two panels of (Fig. 3a), fibrils with higher numbers of protofilament were also observed with an aggregate width of ≥ 116 nm. Fibril length was between ~ 200 and 700 nm. A 2D class average from these fibrils (Fig. 3b) allowed for an increase in the signal to noise ratio, thus enhancing the features in the electron micrographs. Upon closer inspection of the class average (Fig. 3b), the protofilaments display a clear periodicity with a repeat distance of ~ 7–8 nm, suggesting a discrete subunit structure.

**Table 2:** Width measurement of fibrils from patients diagnosed with exfoliation syndrome.

Number of protofilaments	L	M1	M2	M3	H
**Fibril width (nm)**					
One	~ 23	~ 16	~ 13	~ 5	~ 5
Two	–	~ 31	–	~ 11.5	~ 10.5
Three	–	~ 48	–	~ 21	–

Figure 4a shows fibrils that were detected in a sample from a 66-year-old male patient (M2) with moderate XFS. The width of these fibrils was found to be ~ 13 nm (Table 2), with some fibril lengths exceeding 1 μm. Due to the heterogeneity of the fibrils a 2D class average failed to reveal any prominent features. However, the presence of only one density in the 2D class average (Fig. 4b) suggests that the ~ 13 nm fibrils consist of one protofilament only.

Fibrils obtained from sample M3 are similar to those of sample M2, showing filaments with lengths that can exceed 1 μm (Fig. 5a). However, these fibrils had widths between ~ 5 and ~ 21 nm, with a dominant species at ~ 10 nm. The 2D class averages suggested that the 20 nm fibrils are made of two 10 nm protofilaments (Fig. 5c), and although not prominent it is likely that the 10 nm fibrils might contain two 5 nm protofilaments (Fig. 5b).

Fibrils obtained from a 61-year-old male patient (sample L) had a width of ~ 23 nm (Fig. 6a) and a length of ~ 200 nm to ~ 1 μm. In some, but not all, of the 23 nm fibrils two distinct densities were detectable, but this feature was not amplified in the 2D class average (Fig. 6b) indicating a less prominent substructure.

The only XFS patient sample that showed distinct, observable 5 nm protofilaments that were consistent among all fibrils within this sample came from sample H, who was a patient with high stage XFS (Fig. 7a). The fibrils themselves had a diameter of ~ 10.5 nm with a length of between 200 and 500 nm. The two 5 nm protofilaments were made visible in the 2D class average (Fig. 7b). It was interesting to note that this was the only patient in advanced stages of disease.

Twisting is one of the key markers of structural polymorphism in protein fibrils^29^. XFS fibrils from samples XFS-M1, M2, M3, and H showed a helical twist in their structures (arrow, Figs. 3, 4, 5, 7). XFS-H fibril showed the most regular helical pitches (arrow, Fig. 7), however, other XFS fibrils showed less regular twist regions along the fibril axis. The most irregular twist regions were observed in XFS-M2 fibrils (arrow, Fig. 4). To understand the twisted structure of XFS fibrils, their composition and mechanism involving molecular assembly need to be determined. However, twisting is a structural behavior of helical fibrils and amyloids which affect their morphology. It is the result of net interaction arising from two opposing forces; where amino acids tend to twist due to their intrinsic chirality, twisted structures resist further bending stress arising from their elastic distortion^30,34^.

Diameters of XFS protofilaments are as reported in Table 2. Only one type of protofilament was observed in the XFS fibrils obtained from the lens capsule of the patient clinically diagnosed with low XFS. Although in some fibrils of this sample two densities were detected, this structural distinction was not readily recognizable in 2D class average images. In the XFS-M2 sample, protofilaments having a diameter of ~ 13 nm was observed. Samples XFS-M3 and XFS-H both showed two types of protofilaments having similar diameters, whereas, in sample XFS-M3, another type of protofilament with diameter ~ 21 nm was observed. It has been previously shown that the diameter of XFS protofilaments found on the lens capsule was ~ 10 nm^23^. Moreover, a review suggests that the protofilaments could be in the range of ~ 5 to ~ 9 nm in width^4^. In this study, the protofilaments were identified based on the most basic visible component of the fibril, meaning that, for instance, in the XFS-M1 sample (Fig. 3), three types of fibrils having diameters of 16, 31, and 48 nm, would correspond to one, two, or three protofilaments, respectively (Table 2). This is an important aspect of observed structural variation, where it seems that besides the well-known mechanism of lateral aggregation of protofilaments into mature XFS fibrils, there might be some initial molecular-level assembly processes that could lead to formation of thicker fibrils, presumably without going through lateral aggregation process.

## Conclusions

In this study we evaluated fibrils located on the anterior lens capsules of human eyes for the express purpose of elucidating the extent of their structural diversity. Electron micrographs of fibrils from only five patients revealed significant diversity in fibril morphologies. It is striking that given a small sample size such diversity in fibril structure was noted. These results may suggest different phenotypes or clinical subtypes of XFS, however, additional analyses would be required to resolve this question and reveal more detailed information about unknown aspects of XFS fibrils. Further classification including statistical distribution of structural sub-populations will require significantly more samples. Nevertheless, even with a small sample size it was apparent that fibrils are comprised of protofilaments of various diameters and banding patterns.

Fibril polymorphisms are considered an important facet of other fibril-related diseases, but is largely overlooked when considering XFS. For instance, two distinct fibrillar structures were found in samples extracted post-mortem from the brains of two Alzheimer’s disease patients with different clinical histories. Autopsy revealed that both patients had developed characteristic Aβ plaques and neurofibrillary tangles of Alzheimer’s disease, however, different fibril structures suggests that this structural diversity might be correlated with variations in disease development^31^. That said, it is also obvious that fibrils associated with Alzheimer’s, for example, interact with a vastly different environment, viz., cell dense tissue compared to XFS materials located on the anterior lens capsule. However, XFS fibrils are located throughout the anterior segment and within the Schlemm’s canal wall i.e. environments that range from cell free to cell dense. It has been shown that XFS materials are composed of fibrils of varying diameters and lengths^4,32^. However, certain types of fibrils with dominant structural features in different patients have not been observed before.

Taking amyloid fibrils as a basis, polymorphisms are thought to reflect fibril structures, including: variations in the number of protofilaments, differences in the relative orientation of protofilaments, and differences in the internal conformation of the protofilament^33,34^. Furthermore, structural variations of ex vivo amyloid fibrils may be influenced by constituent proteins and fibrillogenesis conditions^35^. Considering these factors, further studies on XFS fibrils obtained from different patients may reveal possible links between precursor proteins and dominating fibrillar ultrastructures, which may shed light on disease progression.

## Methods

### Sample collection

All human tissue samples were collected and all experimental protocols were approved according to protocols approved by the Research Ethics Boards of the University of Alberta and University of Calgary. All methods were carried out in accordance with relevant guidelines and regulations. Informed consent was obtained from all subjects or, if subjects are under 18, from a parent and/or legal guardian. The exclusion criteria included eyes with previous severe trauma or infrared radiation exposure, history of diabetes mellitus, and patients with previously reported amyloidosis. Tissue samples were transferred and kept at 4 °C in BSS intraocular irrigation solution (Alcon). Lens capsules used for microscopy studies of XFS fibrils on the tissue were preserved in fixative buffer (2.5% glutaraldehyde, 4% paraformaldehyde in 0.1 M phosphate buffer, pH 7.4) prior to processing. Lens capsules used for removing XFS materials were washed with ultrapure water (Milli-Q) to remove excess salts prior to processing. Capsules were placed on the coverslip in 25 µl of 1 mM sodium azide and XFS materials were removed through a light abrasion of the surface with the side of a gel loading pipette tip (GELoader, epT.IPS, 20 μL, Eppendorf, Germany). For light microscopy observation, tissue sections were stained with Richardson’s stain (0.1% methylene blue in 0.1% sodium borate, 0.1% azure II).

### Scanning electron microscopy (SEM) of lens capsule having XFS material

Upon washing with 0.1 M phosphate buffer, the lens capsule was dehydrated in a graded series of ethanol with 20% increase in concentration every 15 min followed by hexamethyldisilazane (HMDS) treatment, 25% increments every 30 min. Sample was allowed to air dry overnight, and subsequently sputter-coated with gold–palladium film. SEM images were obtained using a Philips FEI-XL30 scanning electron microscope (FEI Company, CA, USA).

### Transmission electron microscopy (TEM) of XFS deposits on lens capsule

After washing with 0.1 M phosphate buffer, lens capsule was dehydrated through a graded ethanol series. Sample was then infiltrated with a mixture of ethanol and Spurr's resin overnight, followed by 100% Spurr's resin incubation. Thin sections were prepared using an ultramicrotome (Reichert-Jung Ultracut-E, Vienna, Austria) and stained with 4% uranyl acetate solution. Electron micrographs were captured using a Philips–FEI transmission electron microscope (Morgagni-268, Hillsboro, USA) operating at an acceleration voltage of 80 kV.

### TEM of isolated XFS materials

Negative stain electron microscopy was used to assess the different fibrillar deposits of patient-derived XFS samples. XFS materials were removed from the anterior lens capsule surface as described above in sample collection section. XFS patients were classified as having low, moderate, or heavy deposits. 6 μl of each sample was loaded onto a glow-discharged 400 mesh carbon-coated copper grid (Ted Pella Inc. Redding, CA). Typically, samples were adsorbed onto the grid surface for 2 min after which excess solution was removed by blotting with Whatman paper. Grids were then washed briefly and negatively stained using freshly filtered 2% uranyl acetate (UA) or 2% phosphotungstic acid (PTA) solutions. The negatively stained samples were analyzed with a FEI Tecnai G20 electron microscope (FEI Company). An acceleration voltage of 200 kV was used to record micrographs on an Eagle 4 k × 4 k CCD camera (FEI Company).

### Two-dimensional (2D) class averages of the fibril segments

Electron micrographs showing well-defined, isolated fibrillar structures were used for image processing to enhance the signal-to-noise ratio of the fibril ultrastructure. Isolated fibril images were selected and segmented along the fibril axis into 50% overlapping boxes of 200 × 200 pixels (64 nm × 64 nm) using EMAN’s boxer program^36^. Class averages were calculated using the EMAN’s startnrclasses program^36^.

## Data Availability

The data that support the findings of this study are available from the corresponding author upon reasonable request.
